# Making continuous outcomes meaningful to clinicians

**DOI:** 10.1186/1745-6215-12-S1-A66

**Published:** 2011-12-13

**Authors:** Janet L Peacock, Odile Sauzet, Sally M Kerry

**Affiliations:** 1 Division of Health and Social Care Research, King’s College London, SE1 3QD, UK; 2 Public Health Sciences and Medical Statistics, University of Southampton, Southampton, SO16 6YD, UK; 3 Blizard Institute, Barts and The London School of Medicine and Dentistry, Queen Mary University of London, London, E1 2AB,UK

## Objectives

Dichotomizing continuous data prior to analysis is rarely a good idea as power is lost. However expressing results as a difference of means is not always meaningful for clinicians; small differences in means may seem clinically unimportant while the same study results expressed as a difference/ratio of proportions or number needed to treat might reveal an effect that is considered worthwhile. We therefore sought an estimate based on comparing proportions that gives the same p value as the corresponding comparison of means.

## Method

We considered the proportion above a chosen cut-point of a Normal distribution as a function of the mean and standard deviation. This was used to derive the standard error and confidence interval (CI) for a difference in proportions that maintains the power of the corresponding comparison of means. This therefore provides the difference in proportions with a 95% CI that corresponds to a shift in the value of the mean.

To illustrate this method we use published data from a study of teenage motherhood and birth outcomes where 9.7% of 164 young adolescents had a low birthweight LBW, <2500g) baby compared with 3.5% of 423 adults; difference 6.2% 95% CI 1.3 to 11.1%. Using these data we estimated the mean birthweights that would produce these proportions assuming a Normal distribution with standard deviation 500g. Further, we simulated the effects of the method assuming the birthweights were Normally distributed and computed two different 95% CI : i) for the difference in proportion derived from a difference in means as described above, and ii) directly from the difference in proportions. The widths of the two CIs were compared, and the percentage of derived CIs containing the “true” difference of 0.062 (6.2%) was calculated.

## Results

Mean birthweight is estimated as 257g higher in adult mothers. The derived 95% CI for the difference in percentage LBW is 3.5% to 8.9%. Simulation of 10,000 pairs of samples gave mean (SD) ratio of the width of the CI for the difference in proportions, direct/derived, of 2.09 (0.12). In approximately 6% of cases, the 95% CIs for proportions derived from the difference in means did not contain the expected value 0.062 (6.2%).

## Conclusions

A difference in proportions with 95% CIs can be calculated for continuous outcomes without the loss of power usually associated with dichotomising given certain reasonable assumptions. This methodology provides estimates that are meaningful to clinicians, but retains statistical power.

